# Association between changes in molecular biomarkers of cartilage matrix turnover and changes in knee articular cartilage: a longitudinal pilot study

**DOI:** 10.1186/s40634-019-0179-3

**Published:** 2019-05-03

**Authors:** Heide Boeth, Peter C. Raffalt, Aoife MacMahon, A. Robin Poole, Felix Eckstein, Wolfgang Wirth, Frank Buttgereit, Patrik Önnerfjord, Pilar Lorenzo, Cecilia Klint, Anna Pramhed, Georg N. Duda

**Affiliations:** 1 Julius Wolff Institute, Charité - Universitätsmedizin, Berlin, Germany; 2 Department of Surgery, McGill University, Montreal, Quebec Canada; 3 Institute of Anatomy, Paracelsus Medical University, Salzburg & Nuremberg, Salzburg, Austria; 4 Med. Klinik m. S. Rheumatologie und Klinische Immunologie, Charité - Universitätsmedizin, Berlin, Germany; 5 Department of Clinical Sciences Lund, Lund University, Lund, Sweden; 6 AnaMar AB, Lund, Sweden

**Keywords:** Knee osteoarthritis, Biomarkers, Cartilage degradation, Volleyball, Athletes

## Abstract

**Background:**

An early detection of Osteoarthritis is urgently needed and still not possible until today. The aim of the study was to assess whether molecular biomarkers of cartilage turnover are associated with longitudinal change in knee cartilage thickness during a 2 year period in individuals with increased risk of developing knee osteoarthritis. A secondary aim was to assess whether prior knee injury or subjective patient-reported outcomes at baseline (BL) were associated with articular cartilage changes. Nineteen volleyball players (mean age 46.5 ± 4.9 years, 47% male) with a 30-year history of regular high impact training were recruited. The serum biomarkers Cpropeptide of type II procollagen (CPII), cartilage oligomeric matrix protein (COMP), collagenase generated carboxy-terminal neoepitope of type II collagen (sC2C), cartilage intermediate layer protein 2 (CILP-2), and the urine biomarkers C-telopeptide of type II collagen (CTX-II) and collagenase-generated peptide(s) of type II collagen (C2C-HUSA) were assessed at BL and at 2 year follow up (FU). Femorotibial cartilage thinning, thickening and absolute thickness change between BL and FU was evaluated from magnetic resonance imaging. Subjective clinical status at BL was evaluated by the International Knee Documentation Committee Subjective Knee Form and the Short-Form 36 Physical Component Score.

**Results:**

CILP-2 was significantly higher at FU and linearly associated with the absolute cartilage thickness change during the experimental period. Prior injury was a predictor of increased absolute cartilage thickness change.

**Conclusion:**

Measuring the change in the cartilage biomarker CILP-2 might be a valid and sensitive method to detect early development of knee osteoarthritis as CILP-2 appears to be related to cartilage thickness loss in certain individuals with increased risk of developing knee osteoarthritis. Prior knee injury may be predictive of increased articular cartilage thickness change.

## Background

Using current standard diagnostics, i.e. radiographic assessment, knee osteoarthritis (OA) is diagnosed at a relatively late stage of the disease when the knee joint is characterized by substantial cartilage changes (e.g. narrowing of the knee joint space and osteophyte formation). By this time, patients often suffer from functional impairments and pain (Kraus et al., 2011). Use of conservative treatment may have limited impact due to the severity of the disease at this point and surgery is often required (Buttgereit et al., 2015). Recent observations of young athletes with high risk of developing knee OA (Boeth et al., 2017a) emphasize the importance of valid, reliable and sensitive methods to detect development of knee OA prior to presentation of severe structural pathology (Garnero et al., 2000, Guermazi et al., 2011, Roemer and Guermazi 2014).

While the progression of OA may be driven by cartilage factors in some patients, it may be driven by bone or inflammation associated factors in others (Buttgereit et al., 2015). Metabolic alterations in joint tissues associated with different stages of incident OA involve changes in both the synthesis and degradation of skeletal matrix molecules (Kong et al., 2006, Cibere et al., 2009). Previous observations suggest that several molecular biomarkers of cartilage turnover may be helpful in identifying individuals at risk of joint degradation (Vignon et al., 2001, Boeth et al., 2017a, b, Kraus et al., 2017). The serum cartilage type II collagen synthesis biomarker carboxy-propeptide of type II procollagen (CPII) is inversely associated with risk of having radiographically defined knee OA (Nelson et al., 1998, Cibere et al., 2009). In addition, serum levels of cartilage oligomeric matrix protein (COMP), which binds and stabilizes type II collagen fibers, are positively associated with the severity of radiographically assessed knee OA (Clark et al., 1999, Vilim et al., 2001, Rousseau and Delmas 2007). When assessed by magnetic resonance imaging (MRI), knee joint degeneration and OA are associated with serum levels of both COMP and the collagenase generated carboxy-terminal neoepitope of type II collagen (C2C) (King et al., 2004, Hunter et al., 2007). Furthermore, the C-telopeptide of type II collagen (CTX-II), which is often used as an urine biomarker of articular cartilage degradation, is elevated in OA patients compared to controls (Christgau et al., 2001) and associated with the risk (Cibere et al., 2009) and severity of radiographically defined OA (Christgau et al., 2001, Reijman et al., 2004, Jordan et al., 2006), as well as with MRI diagnosed knee cartilage defects (Ding et al., 2005). The collagenase-generated peptide of human type II collagen (C2C-HUSA) assay is predictive of early onset knee OA and its progression (Poole et al., 2016). Finally, serum levels of cartilage intermediate layer protein 2 (CILP-2) have been observed to be reduced in a model of murine OA (Bernardo et al., 2011). The fact that the intermediate zone of healthy adult articular cartilage is particularly enriched with this protein, suggests that changes in CILP-2 may be associated with the progression of arthritis.

While most previous studies on the role of molecular biomarkers in knee cartilage changes have included patients with radiographically diagnosed knee OA, only few studies have investigated this relationship in individuals with an increased risk of developing OA, such as athletes performing high impact sports (Roos et al., 1995, Matsumoto et al., 1997, Creighton et al., 2001, O'Kane et al., 2006, Hoch et al., 2012, Mateer et al., 2015, Boeth et al., 2017b). To our knowledge no previous study has investigated the longitudinal association between molecular biomarkers of cartilage turnover and cartilage thickness changes in such at risk population. In a previous study, we observed significant decrease in knee joint cartilage thickness across a 2 year period in adult former volleyball athletes (Eckstein et al., 2014). However, for this population the potential for using molecular biomarkers of cartilage turnover as predictors of knee cartilage turnover and the possible future development of knee OA is unknown.

The primary aim of the present study was to assess whether cartilage biomarkers were associated with knee cartilage thickness change during a 2 year period in individuals with increased risk of developing knee OA. A secondary aim was to assess whether prior knee injury or subjective patient-reported outcomes at baseline (BL) were associated with cartilage thickness changes. We included athletes with a rigorous training history of high impact activities as a model for individuals with a high risk of developing knee OA (Kujala et al., 1994).

## Methods

### Subjects

Nineteen adult former national level volleyball players (10 females/9 males) over the age of 40 years were included in the present study (Table 1) after giving written informed consent to the experimental procedure, which was approved by the local ethics committee (EA2/055/10) and in accordance with relevant guidelines and regulations. The subjects were assessed at BL and after a 2 year follow up (FU) period. All subjects had a history of participating in volleyball at a German national level since their adolescence and had completed structured volleyball training at least twice a week throughout their career. No instructions were given to alter the intensity or load of the training schedule during participation in the study. At the time of inclusion, all subjects were evaluated for knee injuries that required surgical intervention prior to the study. Exclusion criteria were injuries within 1 year prior to BL. In total, 8 of the included subjects had prior knee surgery greater than 1 year prior to BL. Of the males, 1 had a lateral meniscectomy, 1 had lateral and medial meniscectomies and articular cartilage debridement, 1 had other knee surgery, and 1 had cartilage debridement and meniscus surgery. Of the females, 1 had nerve transection and cartilage debridement, 1 had knee arthroscopy, 1 had meniscus surgery, and 1 had a medial meniscectomy. None of the included subjects were radiographically diagnosed with knee OA. All experiments were performed in accordance with relevant guidelines and regulations.

**Table 1:** Subject demographics (*N* = 19)

	Mean ± standard deviation
Age (years)	46.5 ± 4.9
Body mass (kg)	82.3 ± 16.2
Body height (cm)	183 ± 8.9
BMI (kg/m^2^)	24.3 ± 2.9

### Clinical outcome scores

To assess the subjective clinical status at BL, the subjects completed the Subjective Knee Form of the International Knee Documentation Committee (IKDC) and the Short-Form 36 (SF-36). The IKDC is a validated patient-rated outcome measure that evaluates symptoms, function, and sports activity in patients with knee problems (Irrgang et al., 2001). The SF-36 is a validated metric for general health outcomes (Patel et al., 2007). Only the Physical Component Score (SF-36 PCS) was assessed. For each outcomes measure, scores range from 0 to 100 with a lower score indicating greater disability.

### Molecular biomarkers analyses

Blood samples were obtained following an overnight fast and urine samples were collected on the second morning void. Samples were collected at both BL and FU after MRI scans were completed, prior to which the subjects had been inactive for 45 min. Due to an inadequate blood sample at BL in one subject, serum samples were obtained from 18 subjects at BL and 19 subjects at FU. Urine samples were obtained from all subjects at both BL and FU. Serum and urine samples were immediately stored at − 70 °C until testing. The samples were frozen on dry ice and transported to AnaMar AB (Lund, Sweden) and IBEX Pharmaceuticals (Montreal, Quebec, Canada) for testing.

The analysis of serum cartilage molecular biomarkers involved the following assays: COMP (expressed in U/L), CILP-2 (expressed in ng/mL), sC2C (expressed in ng/mL), and CPII (expressed in ng/mL). Urine biomarkers C2C-HUSA and CTX-II are expressed as ng/mmol of creatinine. CTX-II was supplied by Immunodiagnostic Systems Ltd., Boldon, UK. At IBEX Pharmaceuticals, all samples were assayed in duplicate for sC2C, CPII, C2C-HUSA and CTX-II and urine creatinine. At AnaMar AB, all the samples were assayed in duplicate for COMP and CILP-2. All subjects were randomly assigned to the assay plates. Both time points for each subject were always assayed on the same plate. All analyses were conducted blinded, without prior knowledge of the identity of individual samples. Commercially available assays (all assays except CILP-2) were performed as directed by the manufacturer in accordance with their published guidelines that accompany the assay kits.

### Serum cartilage matrix and degradation biomarkers

COMP was measured using a sandwich enzyme-linked immunosorbent assay (ELISA) (AnaMar AB), CILP-2 using an in-house research competitive ELISA (AnaMar AB), and the cartilage collagen degradation biomarker C2C using a competitive inhibition ELISA (IBEX Technologies).

### Serum cartilage collagen synthesis biomarker

CPII was assayed using a competitive inhibition ELISA (IBEX Technologies).

### Urine cartilage type II collagen degradation biomarkers

C2C-HUSA was assessed using a new sandwich assay (IBEX Technologies,) (Poole et al., 2016) and CTX-II was assessed using a competitive assay (CartiLaps; IDS). The level of creatinine was measured using an enzymatic colorimetric kit (QuantiChrom™; BioAssay Systems, Hayward, CA, USA). Additionally, to assess the relative balance between type II collagen matrix synthesis and degradation, the following ratios were calculated: sC2C/CPII, C2C-HUSA/CPII, and CTX-II/CPII (Nelson et al., 1998, Cibere et al., 2009).

All assays, with the exception of CILP-2, had an inter-assay variability of 2–3%, intra-assay variability of 2–4%, and recovery of 93–116% (for further details please visit the websites of the manufacturers at www.ibex.ca). The CILP-2 competitive assay had an intra-assay variation of 11.1% and the inter-assay variations for high control was 20.7% and for low control 22.2%. For the competitive CILP-2 assay, microtitre plates (Costar high binding 9018) were coated with a 61 amino acid long synthetic polypeptide from the CILP2 domain 1 (V^551^-D^611^ (UniProt Q8IUL8) Schafer-N, Copenhagen, Denmark). Plates were incubated at room temperature overnight. Plates were washed with PBS-Tween (PBS-T, Medicago) 4 times and incubated with block solution (1% Probumin from Millipore in PBS-T) for 2–3 h. Standard (range 100 ng/ml-0.14 ng/ml) and athlete serum samples were diluted 1:10 in conjugate buffer (Medicago 25–0142+ 0.1 mg/ml goat IgG from Sigma) and pre-incubated with HRP-coupled anti-CILP-2 antibody (polyclonal affinity-purified goat antibody (Capra Science, Sweden) against the coating CILP-2 peptide) on non-binding plates (U96 PP 0.5 ml, Natural, Thermo Scientific) for 1 h. The blocked plates were washed with PBS-T 4 times and the pre-incubated sample was transferred to the freshly washed plates followed by further incubation at room temperature for 2 h. After washing with PBS-T (6 times) TMB substrate (Medicago) was added and the signal allowed developing for 20 min protected from light. The reaction was stopped by the addition of 0.5 M H_2_SO_4_ before reading the plates at 450 nm. The CILP-2 assay standard, a 16 kDa protein (His6tagged aa C^206^-P^246^ merged with V^551^-D^611^) was produced in *E. coli* and IMAC/Ni^2+^ column purified. Standard curve was produced by spiking standard protein to non-detecting CILP-2 serum.

### Analysis of articular cartilage thinning and thickening

MRI of the dominant knee was performed at BL and FU. A sagittal 3D VIBE sequence with water excitation (1.5 mm slice thickness; 0.31 mm in-plane resolution, repetition time = 14.6 ms, echo time = 6.5 ms, flip angle = 20°) was used for quantitative analysis of articular cartilage thickness. MRI analysis was performed by an expert reader with 12 years of experience in musculoskeletal image analysis who was blinded to the acquisition order. Segmentation of the subchondral bone and cartilage surface area of the medial and lateral tibia and weight-bearing medial and lateral femoral condyle was performed as described earlier, with all segmentations being quality-controlled by an expert reader (Eckstein et al., 2014). Overall femorotibial changes in articular cartilage thickness between BL and FU were determined from 16 femorotibial subregions, with the location-independent change (thinning and thickening) scores being reported (Wirth and Eckstein 2008, Wirth et al., 2016, Eckstein et al., 2017). The absolute change in cartilage thickness was computed from the changes observed across 16 femorotibial cartilage subregions within each knee by summing the absolute subregional changes (i.e. both greater increase and decrease in subregional cartilage thickness result in a greater absolute thickness change) (Eckstein et al., 2015a, Eckstein et al., 2017).

### Statistics

In order to fulfil the primary aim of the study, two steps were conducted. First, the differences between BL and FU values of the four serum and the two urine biomarkers and the three ratios (sC2C/CPII, C2C-HUSA/CPII, and CTX-II/CPII) were established using paired Student’s t-test. In case of a significant difference between BL and FU, effect size was calculated using Cohen’s *d* (Rosenthal 1991). The effect size was considered large when *d* = 0.8, medium when *d* = 0.5 and small when d = 0.2. Secondly, variables with a significantly different FU value compared to the BL were included in a linear regression analysis to establish the relationship between changes in molecular biomarkers and absolute (location independent) cartilage thickness change. To fulfill the second aim of the study, a backward stepwise multiple linear regression analysis (removal criteria of *p* ≥ 0.10) was performed with total cartilage change as a dependent variable and prior injury requiring surgery (yes/no), IKDC score and SF-36 PCS score as independent variables. Assumptions of normal distribution, linearity and homoscedasticity of the total cartilage change variable were confirmed after visual inspection of the distribution histogram and a plot of the standardized residuals as a function of the standardized predicted values. Level of significance was set at 0.05. All statistical calculations were performed in SPSS (IBM SPSS Statistics, version 24, 2016, USA).

## Results

**Fig. 1:**
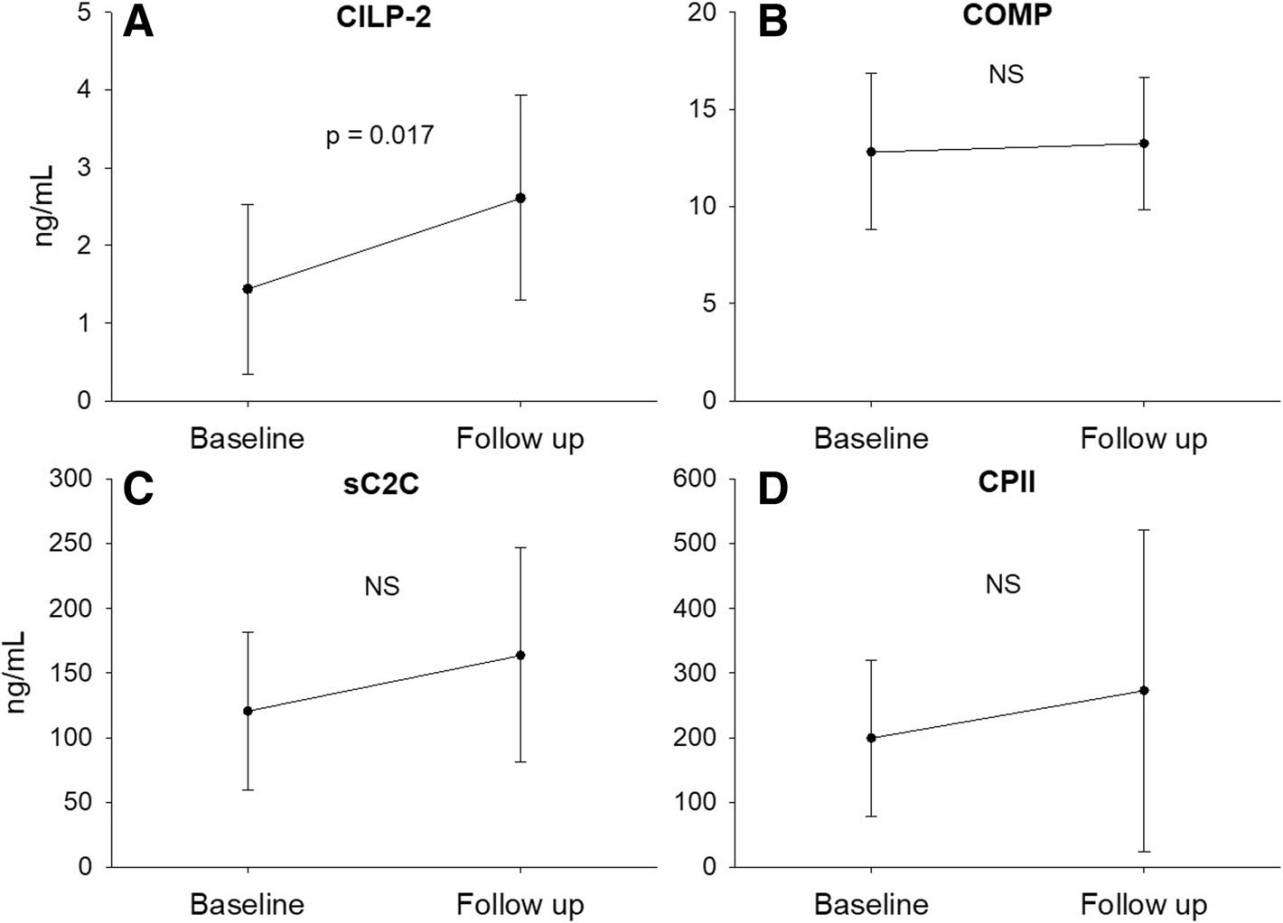
Mean ± standard deviation levels of serum (CILP-2 (**a**), COMP (**b**), sC2C (**c**) and CPII (**d**)) biomarkers at baseline and 2 years follow up. NS: no significant difference between time points

**Fig. 2:**
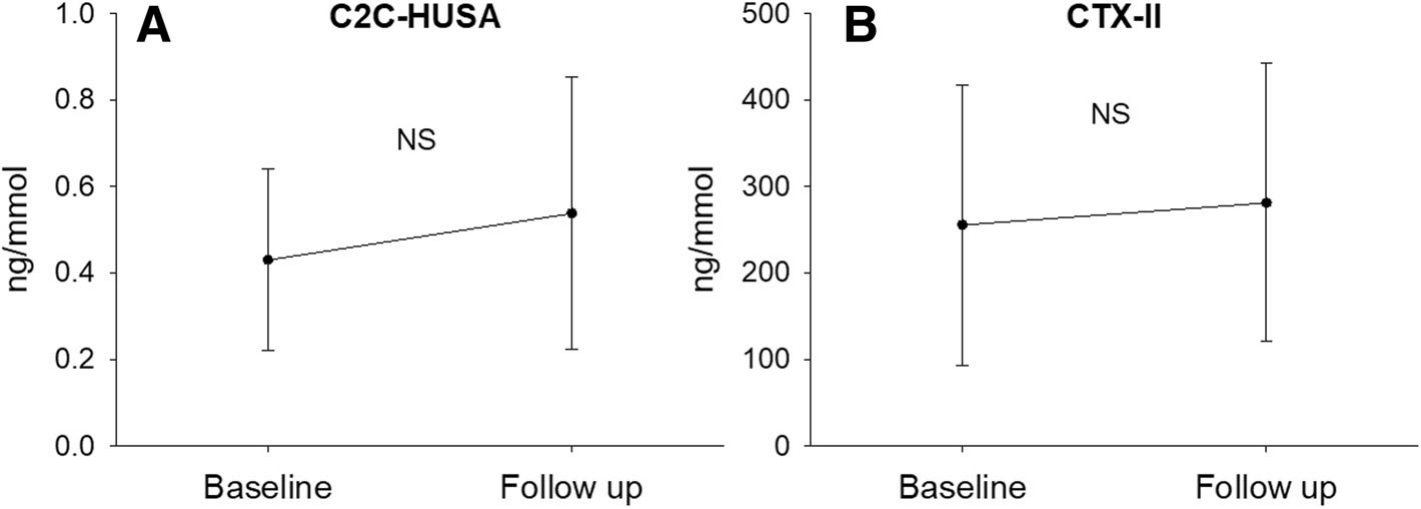
Mean ± standard deviation levels of urine (C2C-HUSA (**a**) and CTX-II (**b**)) biomarkers at baseline and 2 years follow up. NS: no significant difference between time points

**Fig. 3:**
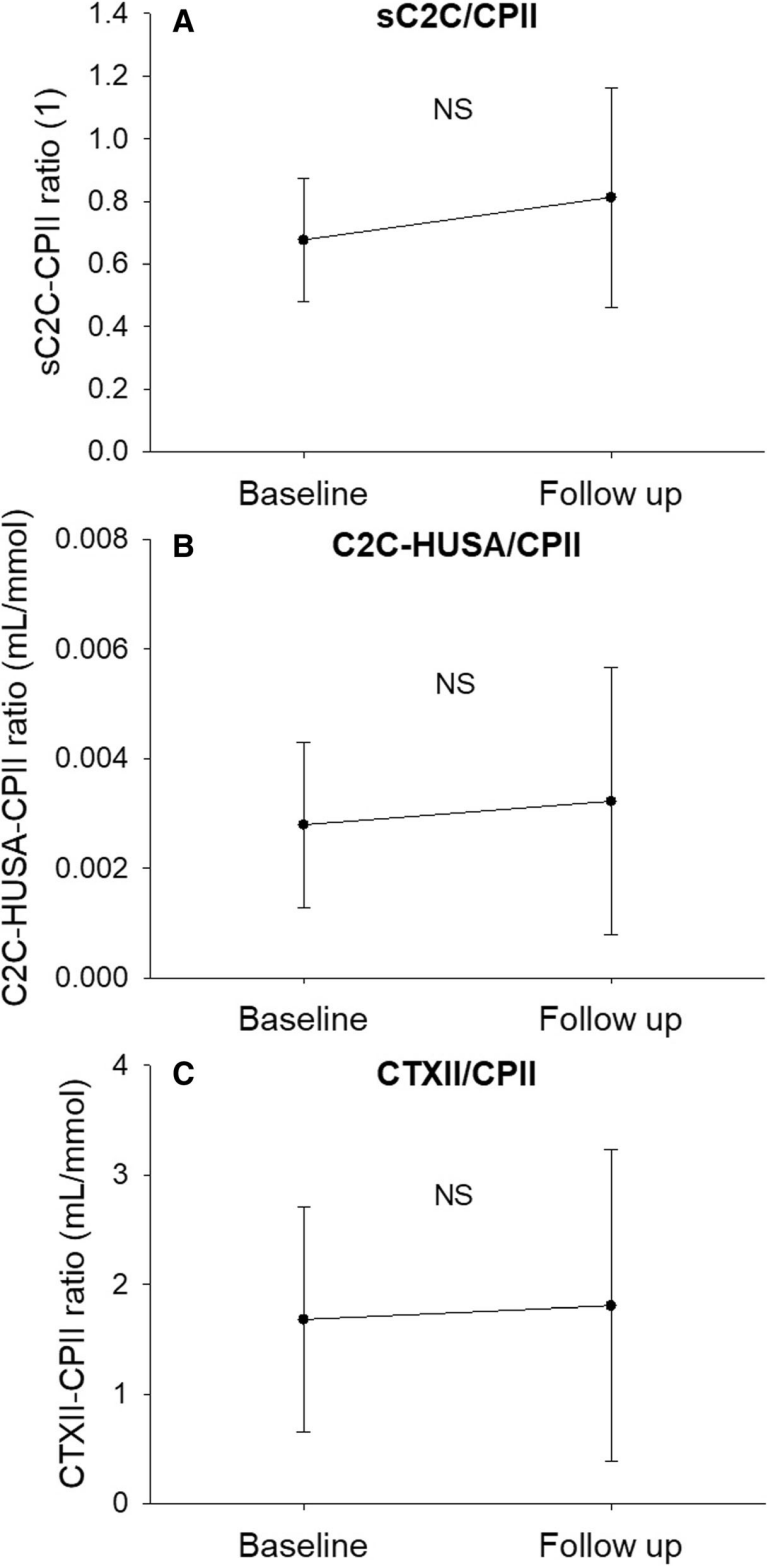
Mean ± standard deviation ratios between molecular biomarkers of cartilage degradation and synthesis (sC2C/CPII (**a**), C2C-HUSA/CPII (**b**), CTXII/CPII (**c**)) at baseline and 2 years follow up. NS: no significant difference between time points

**Fig. 4:**
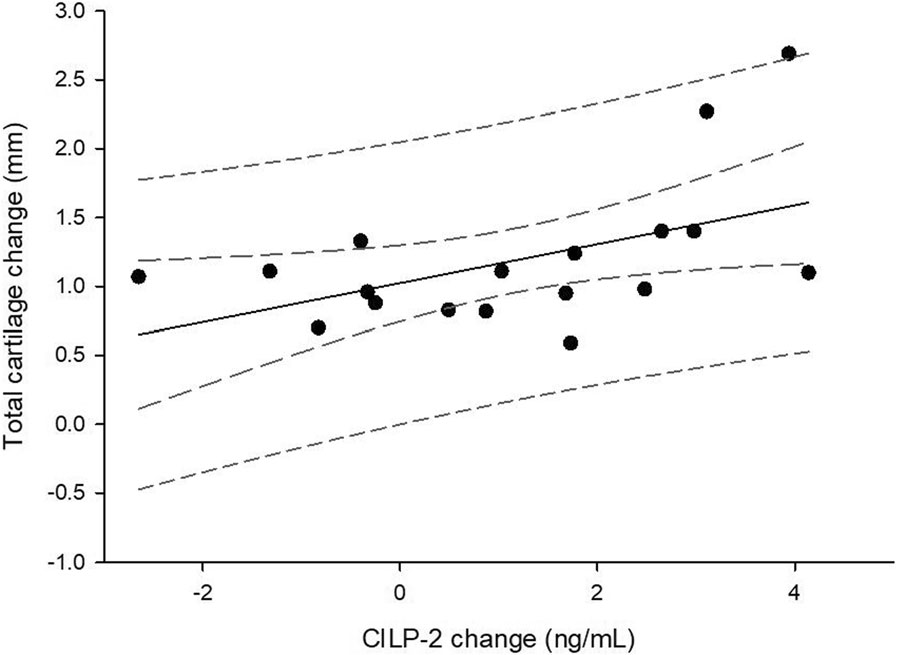
Linear regression analysis plot of total cartilage change as a function of change in CILP-2. Dashed lines are ±95% confidence and 95% prediction lines

**Fig. 5:**
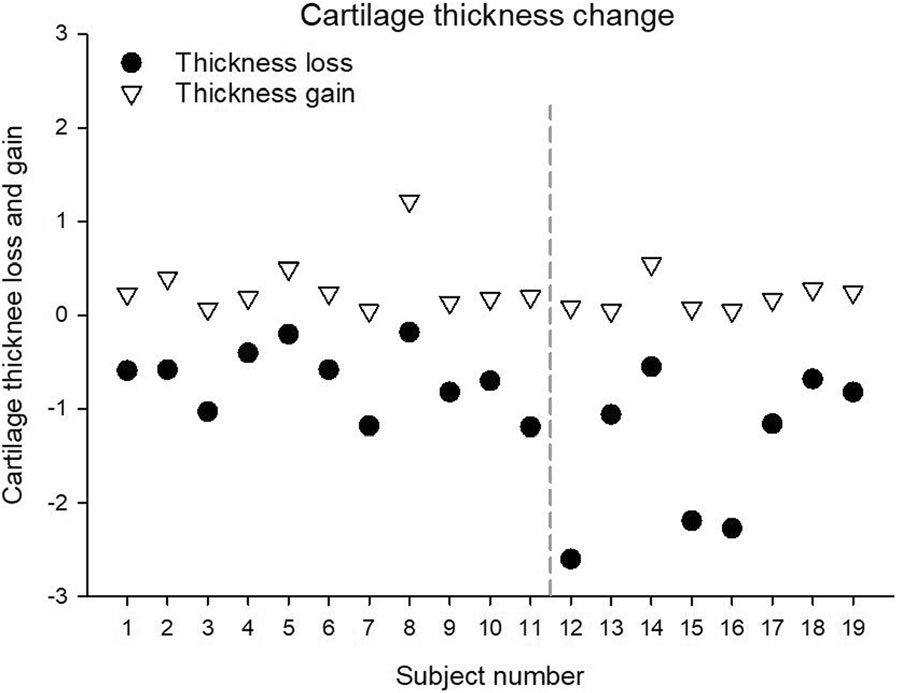
Cartilage thickness changes for all subjects. Positive values indicate cartilage thickness gain and negative values indicate cartilage thickness loss. The total cartilage change corresponds to the absolute vertical distance between the triangle and circle for each subject. The vertical dashed line separates subjects without previous knee injury (to the left of the line, subject 1–11) and subjects with previous knee injury (to right of the line, subject 12–19)

**Fig. 6:**
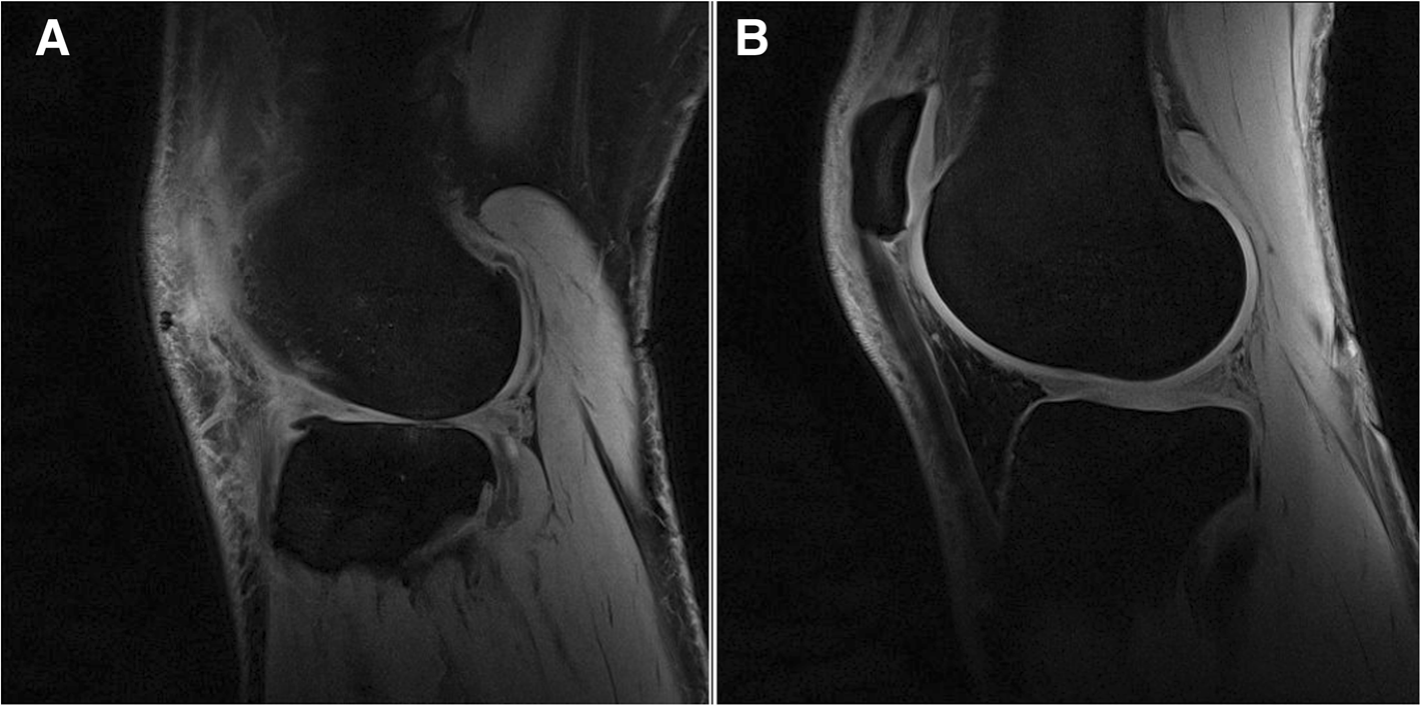
Lateral compartment of male participants with **a** full thickness cartilage damage of the lateral femur and tibia and **b**) without cartilage lesions depicted using sagittal 3D VIBE MRI

When comparing serum and urine biomarkers at BL and FU, CILP-2 was significantly lower at BL (Fig. 1a, *p* = 0.017, effect size = 0.60). No other significant changes in molecular biomarkers from BL to FU were observed (Figs. 1b-d and 2). No significant differences were observed in the degradation/synthesis ratios between BL and FU (Fig. 3). The linear regression analysis revealed a significant linear relationship between CILP-2 change and absolute cartilage thickness change (Fig. 4, F(1,16) = 5.557, *p* = 0.031, r = 0.508, r^2^ = 0.258, adj. r^2^ = 0.211, unstandardized B coefficient (95% confidence interval) = 0.141 (0.014–0.268), standardized β coefficient = 0.508). The absolute contribution of cartilage thickness loss and gain to the total cartilage change for each subject is presented in Fig. 5. Cartilage thickness loss was the largest contributor to the total cartilage change for the majority of subjects. Figure 6 shows full thickness cartilage damage of one adult subject (A) and another adult subject without any cartilage lesions (B) using sagittal 3D VIBE MRI.

The BL mean ± SD IKDC and SF-36 PCS score were 94.3 ± 8.6 and 53.4 ± 2.5, respectively. The stepwise multiple regression analysis removed first the IKDC and then the SF36 variable from the model leaving only previous injury requiring surgery as a predictor of total cartilage changes. This model was significant (F(1,17) = 7.140, *p* = 0.016, r = 0.54, r^2^ = 0.296, adj. r^2^ = 0.254, unstandardized B coefficient (95% confidence interval) = 0.613 (0.129–1.097), standardized β coefficient = 0.544).

## Discussion

This study is the first to investigate long-term changes in cartilage molecular biomarkers in middle aged individuals with increased risk of developing knee OA. Serum and urine biomarkers of cartilage degeneration and synthesis were included to provide potential associations between longitudinal changes in molecular biomarkers and knee cartilage thickness change. This study used volleyball players with a considerable history of high impact training as a model for a high-risk group. None of the included subjects were radiographically diagnosed with knee OA at the time of the study. However, it has previously been suggested that high level athletes in weight bearing sports have an increased risk for developing OA in lower extremity joints (Kujala et al., 1994). Using this experimental model, our primary aim was to assess whether molecular biomarkers of cartilage turnover were associated with knee cartilage thickness change during a 2 year period. We observed that the collagen degeneration serum biomarker CILP-2 was significantly increased with a medium to large effect size at the end of the 2 year period, and that this change was linearly related to the increase in total cartilage change assessed by MRI. Approximately 21% of the variation in total cartilage change could be explained by the variation in the change of CILP-2. Furthermore, for the majority of subjects, the loss of cartilage thickness was greater than the gain across all subregions. The secondary aim of the study was to assess whether prior injury requiring knee surgery or subjective patient-reported outcomes were associated with cartilage changes. We observed that the presence of previous injuries could be a predictor for the total cartilage change such that individuals with prior injuries can be expected to have a higher level of cartilage change.

The positive association between changes in CILP-2 and total cartilage thickness change, which mainly represented articular cartilage loss, suggests that increasing levels of this biomarker coincide with structural progression towards knee OA. Furthermore, since CILP-2 is found in articular cartilage and meniscus (Bernardo et al., 2011), it could be speculated that this biomarker may be used to detect articular cartilage thickness change following synovial tissue damage from long term regular high-impact activity (e.g. volleyball). CILP-2 changes in OA have been little investigated. Tamm and Vija (2015) observed that patients with radiographically diagnosed tibiofemoral OA had significantly lower levels of CILP-2 compared to healthy controls and that the CILP-2 decreased significantly over a 2 year period in the patients. Similarly, CILP-2 was observed to be downregulated in mice with experimental-induced knee OA when cartilage erosion was apparent (Bernardo et al., 2011). Our data suggest loss of CILP-2 in the tissue as it goes up in serum on a long-term scale in adult athletes of a high-impact sport suggesting release from cartilage. This could be due to the ongoing cartilage remodeling in response to the impact stimulus by the regular training performed by the athletes.

Several previous studies have investigated the association of COMP with the incidence and progression of OA, emphasizing its potential as a biomarker of OA pathology. Dragomir et al. (2002) observed COMP to be positively associated with pre-radiographic symptoms of hip OA but not knee OA. Additionally, studies have observed COMP to be positively associated with the radiographic severity of hip and knee OA (Clark et al., 1999, Vilim et al., 2001, Reijman et al., 2004). Furthermore, several studies have assessed short-term changes in serum levels of COMP in athletes. Hoch et al. (2012) investigated changes in COMP in collegiate soccer players during a 3-month soccer season and observed increased levels at mid- and post-season compared to preseason. Neidhart et al. (2000) observed higher BL COMP levels in marathon runners compared to healthy controls, and that COMP levels increased after completing a marathon. Finally, Kersting et al. (2005) investigated the relationship between serum COMP changes and knee cartilage volume changes immediately following 1 hour of running, and observed that an increase in serum COMP was associated with a decrease in knee cartilage volume. While these studies suggest that COMP is positively associated with both acute effects of high impact activities and short-term exposure to high volumes of high impact dominated activities, the present study could not confirm these observations on a long-term scale. The aforementioned studies on soccer players and runners included acute (hours) or short-term (months) increases in activity level and exposure to high impact stimuli, whereas, the present experimental setup did not include a deliberate alteration in activity level during the 2 years. Furthermore, it is likely that the subjects in the present study had experienced considerable higher training loads earlier in their career compared to the period of the study. Thus, the observed cartilage thickness change is presumable primarily a result of the accumulated training throughout the careers of the athletes and not a consequence of alteration in acute or shot-term activity/training exposure. This would suggest that only CILP-2 was able to detect cartilage degradation induced by years-long exposure to high impact training.

The lack of significant difference between the BL and FU ratios for the urine type II collagen degradation biomarker CTX-II and collagen synthesis biomarker ratio suggests that these patients remain in a steady state regarding the balance between type II collagen degradation and synthesis. In a no knee OA, early knee OA and advanced knee OA MRI/biomarker study the uCTX-II/sCPII ratio was both, prognostic of progression of knee OA and able to differentiate between these groups (Cibere et al., 2009). However, this variable has shown not to be an useful indicator of total cartilage change for the population studied here. Previously, the ratio for uC2C/CPII (but not sC2C/sCPII), both not studied here, was also better than individual biomarkers at distinguishing between subjects with pre-radiographically defined and radiographically and MRI defined stages of knee OA (Cibere et al., 2009). The uC2C-HUSA/CPII ratio used here has not yet been studied in knee OA populations.

The scores from the IKDC and SF-36 PCS questionnaires were not found to be predictors of absolute cartilage thickness change. This may be due to a lack of sensitivity in the two questionnaires (e.g. for the IKDC 10 of the 19 subjects achieved a maximum score of 100, demonstrating a strong ceiling effect). These observations question the use of these types of subjective measures to predict cartilage thickness change. In contrast, the regression model revealed that the presence of a previous knee injury is a predictor for the absolute cartilage thickness change observed during the 2 year period. Thus, the included athletes who had suffered from injuries requiring surgery over 1 year prior to BL were more likely to have a higher total cartilage change after 2 years. This is supported by previous studies. Eckstein and colleagues observed an overall thickening of cartilage in the tibiofemoral joint during the first 5 years following ACL rupture, concomitant with localized cartilage thinning in posterior subregions of the medial and lateral tibia (Eckstein et al., 2015b). Although the present study did not assess subregional differences in cartilage change, visual inspection of Fig. 5 indicates that for most of the subjects concomitant overall cartilage thinning and thickening was observed supporting the previous results presented by Eckstein et al. (2015b). Additionally, meniscal tears have been associated with cartilage loss over 2 years in patients with knee OA (Chang et al., 2011). Of the eight injured athletes of the present study, five had meniscal related injuries supporting the link between this type of injury and greater cartilage turnover.

As previously mentioned, the present study relies on the assumption that the included athletes constitute a population with higher risk of developing knee OA. It is unknown at what work load, frequency and intensity that healthy beneficial physical activity becomes a risk factor for development of OA. However, the prevalence of former high level athletes seeking medical treatment for OA later in life has been observed to be significantly higher compared to non-athletes (Kujala et al., 1994). Since the risk of sustaining injuries requiring surgery is likely to increase with the years of participation in volume-high impact training (e.g. competitive sports on national/international level), it is possible that the increased risk of OA in athletes is partly related to them having more severe joint-related injuries compared to non-athletes. Alternatively, it could be speculated that the combination of a long training history including high impact activities and joint injuries requiring surgery increases the risk for developing OA even more than the two parameters separately (Wilder et al., 2002, Teichtahl et al., 2012).

The present pilot study has limitations which need to be taken into account when interpreting the results. The relatively low subject number of patients affects both the statistical power of the study and increases the risk of overfitting of the multiple regression model to the data. Therefore, future studies are needed to increase the number of subjects and the statistical power to confirm these preliminary data. Additionally, caution must be used when extrapolating these findings to other populations considered at high risk of OA, as our subjects were drawn from a specific subpopulation of adults who were prior volleyball athletes. Furthermore, no age-matched control group was included. Thus, it cannot be excluded that the observed changes in CILP-2 and total cartilage change are an effect of aging.

## Conclusions

In conclusion, this present study observed that the cartilage serum biomarker CILP-2 is related to cartilage thickness loss in individuals with increased risk of developing knee OA. Thus, measuring the change in CILP-2 might be a valid and sensitive method to detect early development of knee OA. Additionally, the presence of previous knee injuries requiring surgery was observed to be a predictor for increased total cartilage change.

## Abbreviations

BL: Baseline
C2C: Collagenase generated carboxy-terminal neoepitope of type II collagen
C2C-HUSA: Cartilage degradation-based biomarkers 45 mer collagenase peptide of type II collagen
CILP-2: Cartilage intermediate layer protein
COMP: Cartilage oligomeric matrix protein
CPII: Collagen synthesis marker carboxy-propeptide of type II procollagen
CTX-II: C-telopeptide of type II collagen
ELISA: Enzyme-linked immunosorbent assay
FU: 2-year follow-up
IKDC: International Knee Documentation Committee
MRI: Magnetic Resonance Imaging
OA: Osteoarthritis
PCS: Physical Component Score
SF-36: Short-Form 36

## References

[CR1] Bernardo BC, Belluoccio D, Rowley L, Little CB, Hansen U, Bateman JF (2011). Cartilage intermediate layer protein 2 (CILP-2) is expressed in articular and meniscal cartilage and down-regulated in experimental osteoarthritis. J Biol Chem.

[CR2] Boeth H, MacMahon A, Eckstein F, Diederichs G, Schlausch A, Wirth W, Duda GN (2017). MRI findings of knee abnormalities in adolescent and adult volleyball players. J Exp Orthop.

[CR3] Boeth H, MacMahon A, Poole AR, Buttgereit F, Onnerfjord P, Lorenzo P, Klint C, Pramhed A, Duda GN (2017). Differences in biomarkers of cartilage matrix turnover and their changes over 2 years in adolescent and adult volleyball athletes. J Exp Orthop.

[CR4] Buttgereit F, Burmester GR, Bijlsma JW (2015). Non-surgical management of knee osteoarthritis: where are we now and where do we need to go?. RMD Open.

[CR5] Chang A, Moisio K, Chmiel JS, Eckstein F, Guermazi A, Almagor O, Cahue S, Wirth W, Prasad P, Sharma L (2011). Subregional effects of meniscal tears on cartilage loss over 2 years in knee osteoarthritis. Ann Rheum Dis.

[CR6] Christgau S, Garnero P, Fledelius C, Moniz C, Ensig M, Gineyts E, Rosenquist C, Qvist P (2001). Collagen type II C-telopeptide fragments as an index of cartilage degradation. Bone.

[CR7] Cibere J, Zhang H, Garnero P, Poole AR, Lobanok T, Saxne T, Kraus VB, Way A, Thorne A, Wong H, Singer J, Kopec J, Guermazi A, Peterfy C, Nicolaou S, Munk PL, Esdaile JM (2009). Association of biomarkers with pre-radiographically defined and radiographically defined knee osteoarthritis in a population-based study. Arthritis Rheum.

[CR8] Clark AG, Jordan JM, Vilim V, Renner JB, Dragomir AD, Luta G, Kraus VB (1999). Serum cartilage oligomeric matrix protein reflects osteoarthritis presence and severity: the Johnston County osteoarthritis project. Arthritis Rheum.

[CR9] Creighton DL, Morgan AL, Boardley D, Brolinson PG (2001). Weight-bearing exercise and markers of bone turnover in female athletes. J Appl Physiol (1985).

[CR10] Ding C, Garnero P, Cicuttini F, Scott F, Cooley H, Jones G (2005). Knee cartilage defects: association with early radiographic osteoarthritis, decreased cartilage volume, increased joint surface area and type II collagen breakdown. Osteoarthr Cartil.

[CR11] Dragomir AD, Kraus VB, Renner JB, Luta G, Clark A, Vilim V, Hochberg MC, Helmick CG, Jordan JM (2002). Serum cartilage oligomeric matrix protein and clinical signs and symptoms of potential pre-radiographic hip and knee pathology. Osteoarthr Cartil.

[CR12] Eckstein F, Boeth H, Diederichs G, Wirth W, Hudelmaier M, Cotofana S, Hofmann-Amtenbrink M, Duda G (2014). Longitudinal change in femorotibial cartilage thickness and subchondral bone plate area in male and female adolescent vs. mature athletes. Ann Anat.

[CR13] Eckstein F, Buck R, Wirth W (2017). Location-independent analysis of structural progression of osteoarthritis-taking it all apart, and putting the puzzle back together makes the difference. Semin Arthritis Rheum.

[CR14] Eckstein F, Wirth W, Guermazi A, Maschek S, Aydemir A (2015). Brief report: intraarticular sprifermin not only increases cartilage thickness, but also reduces cartilage loss: location-independent post hoc analysis using magnetic resonance imaging. Arthritis Rheumatol.

[CR15] Eckstein F, Wirth W, Lohmander LS, Hudelmaier MI, Frobell RB (2015). Five-year followup of knee joint cartilage thickness changes after acute rupture of the anterior cruciate ligament. Arthritis Rheumatol.

[CR16] Garnero P, Rousseau JC, Delmas PD (2000). Molecular basis and clinical use of biochemical markers of bone, cartilage, and synovium in joint diseases. Arthritis Rheum.

[CR17] Guermazi A, Roemer FW, Burstein D, Hayashi D (2011). Why radiography should no longer be considered a surrogate outcome measure for longitudinal assessment of cartilage in knee osteoarthritis. Arthritis Res Ther.

[CR18] Hoch JM, Mattacola CG, Bush HM, Medina McKeon JM, Hewett TE, Lattermann C (2012). Longitudinal documentation of serum cartilage oligomeric matrix protein and patient-reported outcomes in collegiate soccer athletes over the course of an athletic season. Am J Sports Med.

[CR19] Hunter DJ, Li J, LaValley M, Bauer DC, Nevitt M, DeGroot J, Poole R, Eyre D, Guermazi A, Gale D, Felson DT (2007). Cartilage markers and their association with cartilage loss on magnetic resonance imaging in knee osteoarthritis: the Boston osteoarthritis knee study. Arthritis Res Ther.

[CR20] Irrgang JJ, Anderson AF, Boland AL, Harner CD, Kurosaka M, Neyret P, Richmond JC, Shelborne KD (2001). Development and validation of the international knee documentation committee subjective knee form. Am J Sports Med.

[CR21] Jordan KM, Syddall HE, Garnero P, Gineyts E, Dennison EM, Sayer AA, Delmas PD, Cooper C, Arden NK (2006). Urinary CTX-II and glucosyl-galactosyl-pyridinoline are associated with the presence and severity of radiographic knee osteoarthritis in men. Ann Rheum Dis.

[CR22] Kersting UG, Stubendorff JJ, Schmidt MC, Bruggemann GP (2005). Changes in knee cartilage volume and serum COMP concentration after running exercise. Osteoarthr Cartil.

[CR23] King KB, Lindsey CT, Dunn TC, Ries MD, Steinbach LS, Majumdar S (2004). A study of the relationship between molecular biomarkers of joint degeneration and the magnetic resonance-measured characteristics of cartilage in 16 symptomatic knees. Magn Reson Imaging.

[CR24] Kong SY, Stabler TV, Criscione LG, Elliott AL, Jordan JM, Kraus VB (2006). Diurnal variation of serum and urine biomarkers in patients with radiographic knee osteoarthritis. Arthritis Rheum.

[CR25] Kraus VB, Burnett B, Coindreau J, Cottrell S, Eyre D, Gendreau M, Gardiner J, Garnero P, Hardin J, Henrotin Y, Heinegard D, Ko A, Lohmander LS, Matthews G, Menetski J, Moskowitz R, Persiani S, Poole AR, Rousseau JC, Todman M (2011). Application of biomarkers in the development of drugs intended for the treatment of osteoarthritis. Osteoarthr Cartil.

[CR26] Kraus VB, Collins JE, Hargrove D, Losina E, Nevitt M, Katz JN, Wang SX, Sandell LJ, Hoffmann SC, Hunter DJ, Consortium OAB (2017). Predictive validity of biochemical biomarkers in knee osteoarthritis: data from the FNIH OA biomarkers Consortium. Ann Rheum Dis.

[CR27] Kujala UM, Kaprio J, Sarna S (1994). Osteoarthritis of weight bearing joints of lower limbs in former elite male athletes. BMJ.

[CR28] Mateer JL, Hoch JM, Mattacola CG, Butterfield TA, Lattermann C (2015). Serum cartilage oligomeric matrix protein levels in collegiate soccer athletes over the duration of an athletic season: a pilot study. Cartilage.

[CR29] Matsumoto T, Nakagawa S, Nishida S, Hirota R (1997). Bone density and bone metabolic markers in active collegiate athletes: findings in long-distance runners, judoists, and swimmers. Int J Sports Med.

[CR30] Neidhart M, Muller-Ladner U, Frey W, Bosserhoff AK, Colombani PC, Frey-Rindova P, Hummel KM, Gay RE, Hauselmann H, Gay S (2000). Increased serum levels of non-collagenous matrix proteins (cartilage oligomeric matrix protein and melanoma inhibitory activity) in marathon runners. Osteoarthr Cartil.

[CR31] Nelson F, Dahlberg L, Laverty S, Reiner A, Pidoux I, Ionescu M, Fraser GL, Brooks E, Tanzer M, Rosenberg LC, Dieppe P, Robin Poole A (1998). Evidence for altered synthesis of type II collagen in patients with osteoarthritis. J Clin Invest.

[CR32] O'Kane JW, Hutchinson E, Atley LM, Eyre DR (2006). Sport-related differences in biomarkers of bone resorption and cartilage degradation in endurance athletes. Osteoarthr Cartil.

[CR33] Patel AA, Donegan D, Albert T (2007). The 36-item short form. J Am Acad Orthop Surg.

[CR34] Poole AR, Ha N, Bourdon S, Sayre EC, Guermazi A, Cibere J (2016). Ability of a urine assay of type II collagen cleavage by collagenases to detect early onset and progression of articular cartilage degeneration: results from a population-based cohort study. J Rheumatol.

[CR35] Reijman M, Hazes JM, Bierma-Zeinstra SM, Koes BW, Christgau S, Christiansen C, Uitterlinden AG, Pols HA (2004). A new marker for osteoarthritis: cross-sectional and longitudinal approach. Arthritis Rheum.

[CR36] Roemer FW, Guermazi A (2014). Osteoarthritis year in review 2014: imaging. Osteoarthr Cartil.

[CR37] Roos H, Dahlberg L, Hoerrner LA, Lark MW, Thonar EJ, Shinmei M, Lindqvist U, Lohmander LS (1995). Markers of cartilage matrix metabolism in human joint fluid and serum: the effect of exercise. Osteoarthr Cartil.

[CR38] Rosenthal R (1991). Meta-analytic procedures for social research.

[CR39] Rousseau JC, Delmas PD (2007). Biological markers in osteoarthritis. Nat Clin Pract Rheumatol.

[CR40] Tamm A, Pramhed A, Klint C, Kumm J, Vija M (2015) Serum levels of CLIP-2 and COMP reflect differently radiographic severity of knee osteoarthritis in middleaged subjects. Clin Chem Lab Med 53 (Suppl 1), S120-S120.

[CR41] Teichtahl AJ, Wluka AE, Wang Y, Forbes A, Davies-Tuck ML, English DR, Giles GG, Cicuttini FM (2012). Effect of long-term vigorous physical activity on healthy adult knee cartilage. Med Sci Sports Exerc.

[CR42] Vignon E, Garnero P, Delmas P, Avouac B, Bettica P, Boers M, Ehrich E, MacKillop N, Rovati L, Serni U, Spector T, Reginster JY (2001). Recommendations for the registration of drugs used in the treatment of osteoarthritis: an update on biochemical markers. Osteoarthr Cartil.

[CR43] Vilim V, Vytasek R, Olejarova M, Machacek S, Gatterova J, Prochazka B, Kraus VB, Pavelka K (2001). Serum cartilage oligomeric matrix protein reflects the presence of clinically diagnosed synovitis in patients with knee osteoarthritis. Osteoarthr Cartil.

[CR44] Wilder FV, Hall BJ, Barrett JP, Lemrow NB (2002). History of acute knee injury and osteoarthritis of the knee: a prospective epidemiological assessment. The Clearwater Osteoarthritis Study. Osteoarthritis Cartilage.

[CR45] Wirth W, Eckstein F (2008). A technique for regional analysis of femorotibial cartilage thickness based on quantitative magnetic resonance imaging. IEEE Trans Med Imaging.

[CR46] Wirth W, S M, Haefner P, Boeth H, Duda G, Eckstein F (2016). Clinical validation of a regulatory compliant software for quantitative analysis of articular cartilage morphology (abstract). Osteoarthr Cartil.

